# Combination of Alpha-Melanocyte Stimulating Hormone with Conventional Antibiotics against Methicillin Resistant *Staphylococcus aureus*

**DOI:** 10.1371/journal.pone.0073815

**Published:** 2013-09-09

**Authors:** Madhuri Singh, Ravisekhar Gadepalli, Benu Dhawan, Kasturi Mukhopadhyay

**Affiliations:** 1 School of Environmental Sciences, Jawaharlal Nehru University, New Delhi, India; 2 Department of Microbiology, All India Institute of Medical Sciences, New Delhi, India; University of Calgary, Canada

## Abstract

Our previous studies revealed that alpha-melanocyte stimulating hormone (α-MSH) is strongly active against *Staphylococcus aureus* (*S. aureus*) including methicillin resistant *S. aureus* (MRSA). Killing due to α-MSH occurred by perturbation of the bacterial membrane. In the present study, we investigated the *in vitro* synergistic potential of α-MSH with five selected conventional antibiotics viz., oxacillin (OX), ciprofloxacin (CF), tetracycline (TC), gentamicin (GM) and rifampicin (RF) against a clinical MRSA strain which carried a type III staphylococcal cassette chromosome *mec* (SCC*mec*) element and belonged to the sequence type (ST) 239. The strain was found to be highly resistant to OX (minimum inhibitory concentration (MIC) = 1024 µg/ml) as well as to other selected antimicrobial agents including α-MSH. The possibility of the existence of intracellular target sites of α-MSH was evaluated by examining the DNA, RNA and protein synthesis pathways. We observed a synergistic potential of α-MSH with GM, CF and TC. Remarkably, the supplementation of α-MSH with GM, CF and TC resulted in ≥64-, 8- and 4-fold reductions in their minimum bactericidal concentrations (MBCs), respectively. Apart from membrane perturbation, in this study we found that α-MSH inhibited ∼53% and ∼47% DNA and protein synthesis, respectively, but not RNA synthesis. Thus, the mechanistic analogy between α-MSH and CF or GM or TC appears to be the reason for the observed synergy between them. In contrast, α-MSH did not act synergistically with RF which may be due to its inability to inhibit RNA synthesis (<10%). Nevertheless, the combination of α-MSH with RF and OX showed an enhanced killing by ∼45% and ∼70%, respectively, perhaps due to the membrane disrupting properties of α-MSH. The synergistic activity of α-MSH with antibiotics is encouraging, and promises to restore the lost potency of discarded antibiotics.

## Introduction

Infections due to methicillin resistant *Staphylococcus aureus* (MRSA) are becoming untreatable and putting tremendous pressure on healthcare systems [1]–[3]. MRSA is the most common cause of infection at a variety of sites including skin and soft tissues, respiratory tract, bloodstream, and prosthetic devices [4]. Beta lactam antibiotics, the wonder drugs of the past, are now no longer effective against these resistant bacteria. Likewise, promising new antibiotics of various classes, recently approved by the Food and Drug Administration (FDA), including linezolid (an oxazolidinone) and daptomycin (a cyclic lipopeptide), could not significantly improve the outcomes of infections caused by MRSA [5], [6]. In fact, emergence of resistance to these new classes of antibiotics may hinder the use of these drugs in the future. With this existing scenario, an effective solution may be the development of combination therapies involving antimicrobial agents with different mechanisms of inhibitory action [7], [8]. For example, pairing of vancomycin with rifampin or gentamicin has been often successful in the treatment of endocarditis caused by MRSA [9]. The potential benefits of combination therapy over single therapy include, reduction in the dose of toxic antibiotics, decreased resistance development, and broader antibacterial activity [10], [11]. In the last three decades, host defense peptides (HDPs), which are key components of innate immunity in a vast array of organisms, have drawn a lot of attention as potential therapeutic agents [12]–[14]. These peptides are modulators of the immune system and evoke effector mechanisms to rapidly stop pathogen proliferation [15]. Moreover, they work synergistically with other HDPs in the host environment [16]. Because of their unique mechanism of targeting microbes and their role in triggering host immunity, HDPs exhibit immense potential to act synergistically with conventional antibiotics. Thus, the pairing of HDPs with other conventional antibiotics or with other HDPs has received greater priority over the pairing of antibiotics alone [17]–[20].

We have earlier established the strong antibacterial activity of alpha-melanocyte stimulating hormone (α-MSH) and its congeners containing C-terminal amino acids against both reference and clinical isolates of *Staphylococcus aureus* (*S. aureus*) [21]–[23]. Subsequently, we confirmed that the staphylocidal action of α-MSH occurred due to depolarization and permeabilization of the bacterial membrane [22], [23]. In the present study, our aim was to explore the *in vitro* synergistic effects of α-MSH with conventional antibiotics. The rationale behind this work was to exploit the membrane permeabilizing property of α-MSH in order to improve the lost antibacterial efficacy of conventional antibiotics. For this purpose, we evaluated the *in vitro* combination effect of α-MSH with five different antibiotics viz., (oxacillin (OX), gentamicin (GM), rifampicin (RF), tetracycline (TC), and ciprofloxacin (CF)) against a clinical MRSA strain which was resistant to all these five antibiotics at high concentrations. We observed a dramatic increment in the staphylocidal effect of each antibiotic even at lower doses when combined with an ineffective dose of α-MSH *in vitro*. These results are very encouraging as combining α-MSH with currently discarded traditional antibiotics might be useful for the treatment of fatal infections due to resistant microorganisms.

## Materials and Methods

### Materials

All the antibiotics viz., oxacillin sodium salt, ciprofloxacin hydrochloride, tetracycline hydrochloride, rifampicin, gentamicin sulfate, synthetic α-MSH were purchased from Sigma-Aldrich (USA). Brain heart infusion (BHI), cation-adjusted Muller Hinton broth (CAMHB), and agar were purchased from HiMedia (India). Scintillation cocktail ‘O’ in toluene was bought from Spectrochem (Mumbai, India). Tricarboxylic acid, MTT (3-(4,5-Dimethylthiazol-2yl)-2,5-diphenyle tetrazolium bromide), trypan blue, tritonX−100 and dimethyl sulfoxide (DMSO) were purchased from Fisher Scientific (Germany). [methyl-^3^H] thymidine, [4,5-^3^H] leucine and [5-^3^H] uridine were purchased from the Board of Radiation and Isotope Technology (BRIT, India). The purity of α-MSH was >97% and its concentration was determined spectrophotometrically (UV-2450 UV-VIS spectrophotometer, Shimadzu). Stocks of all the antibiotics and α-MSH were stored at 4°C.

### Bacterial Strains

The clinical strain of *S. aureus* was isolated from a patient admitted to the All India Institute of Medical Sciences (AIIMS), New Delhi, India with skin and soft tissue infection (SSTI). Ethical approval was obtained from the Institute Ethics Committee, AIIMS, New Delhi, India. *S. aureus* was identified using standard biochemical tests using multiplex PCR. Cefoxitin disk diffusion method and *mecA* PCR was used to characterize the strain as MRSA [24]. Additionally, one prototype ATCC MRSA 33591 and ATCC MSSA 29213 were used to perform quality control as recommended by the Clinical Laboratory Standard Institute (CLSI) [25]. Strains were stored at −80°C in 15% glycerol until sub-cultured onto BHI agar plate followed by secondary culture in BHI broth. The mid log phase grown cells (OD_600 nm_ = 0.5) were used for all the experiments. The cell suspension of desired density was prepared in 10 mM sodium potassium phosphate buffer containing 150 mM NaCl (PBS).

### Molecular Characterization of the Clinical MRSA Strain

The clinical MRSA isolate was processed for staphylococcal cassette chromosome *mec* (SCC*mec*) typing and multilocus sequence typing (MLST) as previously described [24], [26], [27]. The sequences of the seven housekeeping genes (*arcC*, *aroE*, *glpF*, *gmk*, *pta*, *tpi*, and *yqiL*) obtained were compared with the sequences at the MLST website (http://www.mlst.net) to determine the sequence type (ST).

### Minimum Inhibitory Concentration (MIC)

MIC values of all the antibiotics, i.e., OX, RF, GM, CF, and TC were determined by broth microdilution assay following CLSI guidelines using CAMHB [25]. The assay was repeated on three days independently, and a constant MIC value was found. MIC values of OX >2 µg/ml [28], GM >0.25 µg/ml [29], CF >0.25 µg/ml [30], TC >0.25 µg/ml [31], and RF >0.015 µg/ml [28] were considered resistant. MIC value of α-MSH could not be determined as MHB/BHI medium tends to reduce its antibacterial activity. The same inhibitory effect of MHB medium for another cationic antimicrobial peptide, i.e., LL−37 has also previously been reported by Turner et al., [32]. Nevertheless, they had found that the peptide was very active when they performed the killing assays by viable colony count method using PBS buffer [32].

### Minimum Bactericidal Concentration (MBC)

MBC was determined by the killing assay using 10^5^ to 10^6^ CFU/ml of mid log culture as described elsewhere [22], [23], [32], [33]. Briefly, the cells were incubated with each of the selected antibiotic (2 µg/ml to 2048 µg/ml) and α-MSH (2 µg/ml to 160 µg/ml) individually in PBS. The mixture was incubated at 37°C for 2 h and the aliquots were 10 -fold diluted two times serially in PBS (to reduce the drug carry over and to allow accurate colony count) followed by plating on BHI agar plate in triplicate. Viable colonies were counted after overnight incubation and percentage of killing of treated sample was determined with respect to the untreated samples (control). The concentrations at which ≥50%, and ≥90% of cells were killed, were defined as MBC_50_ and MBC_90,_ respectively. MBC values of OX >0.5 µg/ml [28], GM >6.5 µg/ml [34], CF >2 µg/ml [35], TC >3 µg/ml [36], and RF >0.125 µg/ml [37] were considered resistant.

### Synergism Studies

For determining synergism, both the pairing agents (antibiotic and α-MSH) were administered at doses which did not show significant bacterial killing (≥80% cell survival) when used alone. The methodology was similar to the killing assay protocol as described above. Briefly, the cells (10^5^ to 10^6^ CFU/ml) were treated with ineffective doses of each antibiotic alone ranging from 2 to 32 µg/ml and in presence and absence of α-MSH (8 µg/ml). For example, to test synergism between α-MSH and OX, cells were treated with OX alone in the concentration range of 2 to 32 µg/ml and in the presence of 8 µg/ml of α-MSH. An MBC_50_ and MBC_90_ value was calculated for each antibiotic alone and in the presence of α-MSH. A ≥4-fold reduction in MBC_90_ value in the presence of α-MSH was considered a synergistic relation between α-MSH and that antibiotic [28].

### Impact of α-MSH on Bacterial DNA, RNA and Protein Synthesis

To determine the effect of α-MSH on bacterial macromolecule synthesis, whole cell labeling was done with radioactive precursors of DNA, RNA and protein as described elsewhere [38], [39]. In brief, ∼10^8^ CFU/ml of *S. aureus* ATCC 29213 cells were labeled with 1 µci of radioactive precursor of DNA, RNA or protein, respectively; either [methyl-^3^H] thymidine or [5-^3^H] uridine or [4,5-^3^H] leucine. This was followed by treatment with sub-lethal doses of α-MSH (2 µg/ml and 10 µg/ml) for 30 min, 60 min, and 120 min. In addition, 2 µg/ml of each of CF, TC and RF were used as positive controls for DNA, protein and RNA synthesis inhibition, respectively. At selected time points, aliquots were removed from the mixture, added to the chilled 10% tricarboxylic acid (TCA) for 30 min to stop the reaction and filtered through a manifold unit (Millipore). Cells were collected on Millipore filter paper (0.22 µm pore size), dried under infrared light, added to scintillation fluid and the radioactive signal was measured using a Scintillation Counter (Perkin Elmer, USA). Simultaneously, to confirm that the chosen doses of α-MSH were not bactericidal, killing kinetics using the 2 and 10 µg/ml of α-MSH against ∼10^8^ CFU/ml of *S. aureus* ATCC 29213 were performed using duplicate samples prepared separately, without radioactive precursors. Three independent experiments were done in each case.

### Hemolytic Activity of α-MSH

Fresh 5 ml aliquots of blood were collected from Swiss albino mouse in the presence of 2 mg/ml ethylenediaminetetraacetic acid (EDTA) and centrifuged at 400 rpm for 10 min and the red blood cells (RBCs) pellet was collected. The use of animals was approved by the Institutional Animal Ethics Committee of Jawaharlal Nehru University (IAEC-JNU), New Delhi, India. The pellet was washed 3 times in 10 mM PBS buffer, pH 7.4, and re-suspended in 4-fold volumes of the same buffer. 5 µl of this RBC solution was added to 995 µl of PBS buffer containing serially diluted α-MSH (100 pg/ml to 100 µg/ml). The RBC and α-MSH mixture was incubated at 37°C for 1 h or 18 h and centrifuged. To the 200 µl of supernatant 800 µl of PBS buffer was added and absorbance was measured at 413 nm using UV-VIS Spectrophotometer (Shimadzu, Japan). To ensure 100% (positive control) and 0% (negative control) hemolysis, 0.1% tritonX−100 (TX100) and PBS buffer were added, respectively, instead of α-MSH and absorbance at 413 nm was measured. Hemolysis due to the test peptide was determined as described elsewhere [40], % Hemolysis = 100×(absorbance in peptide – absorbance in PBS)/(absorbance in TX100 - absorbance in PBS). The assay was done in duplicate.

### Cytotoxicity Due to α-MSH by MTT Assay

The efficacy of the peptide to induce cell death in a 3T3 mouse fibroblast cell line was determined by MTT assay as described elsewhere [41]. This colorimetric assay is based upon the reduction of yellow tetrazolium dye, MTT, to an insoluble purple colored formazan product by the enzyme succinate dehydrogenase found in metabolically active cells. After treatment with MTT, the cells are solubilized in the presence of organic solvent (i.e., DMSO), and the released, solubilized formazan product is measured spectrophotometrically at 570 nm. Since reduction of MTT can only happen in metabolically active cells, the intensity of formazan is directly proportional to the cell viability. Briefly, a monolayer of 1 day old 3T3 cells (10^5^ cells/ml predetermined by trypan blue staining) were grown in DMEM medium supplemented with 10% fetal bovine serum on 24 well plates placed in a 5% CO_2_ incubator. The cells were washed and re-suspended in PBS buffer and then treated with α-MSH (0.2 µg/ml to 20 µg/ml) for 2 h. Untreated cells in the presence of PBS buffer alone were taken as a negative control (100% intact cells). To the mixture, 20 µl of 5 mg/ml MTT solution was added and incubated in the dark at 37°C for 4.5 h, followed by addition of 200 µl DMSO and further incubation for 1 h. After incubation, 100 µl of this mixture was removed and added to 900 µl of double distilled H_2_O and absorbance was measured at 570 nm against a background (100 µl DMSO+900 µl ddH_2_O). The percentage cytotoxicity was calculated as described elsewhere [41], % of Cytotoxicity = 100×(absorbance of control - absorbance of sample)/(absorbance of control). The above assay was done in triplicate.

### Statistical Analysis

All data was compiled by using mean ± standard deviation calculated for three independent replicates using Microsoft office excel. Difference in mean values among % survival data-sets for various antibiotics alone and in the presence of α-MSH and % survival data-sets for different concentrations of the same antimicrobial agent were measured by one-way Analysis of Variance (ANOVA) using Minitab statistical software [22], [23], and p values ≤0.05 were considered significant.

## Results

### Genotypic Characterization of the Clinical MRSA Strain

The clinical MRSA strain carried a type III cassette, and MLST profiling classified this strain as ST239. The strain is close to UK EMRSA-1 (NCTC11931), and the Brazilian/Hungarian clone (ST239-SCC*mec* III) with allelic profile of 2-3-1-1-4-4-3 based on the DNA sequence of seven housekeeping genes. The predominance of the ST239 clone has been reported previously among Indian nosocomial MRSA strains [24], [42].

### Susceptibility Profiles of *S. aureus* strains to the Selected Antibiotics by Microdilution Assay

MIC values of the selected antibiotics CF, GM, TC, RF and OX against the clinical MRSA isolate as well as ATCC MRSA 33591 and ATCC MSSA 29213 were determined and are presented in Table 1. The ATCC MRSA 33591 strain was resistant to OX (MIC = 128 µg/ml), RF (MIC = 0.5 µg/ml), and TC (MIC = 32 µg/ml), while it was susceptible to both GM and CF. However, the clinical MRSA isolate showed very high MIC values for all antibiotics tested indicating its resistance to both bacteriostatic (TC) and bactericidal (OX, GM, CF, RF) groups of drugs. For example, the MIC values for OX and GM were 1024 µg/ml and 128 µg/ml, respectively, which are 512-fold greater than the values in susceptible bacteria [43]. The rationale behind choosing the clinical MRSA over prototype ATCC MRSA 33591 for the synergistic study was its high resistance towards all the tested antibiotics.

**Table 1:** Minimum Inhibitory concentration (MIC) values of selected antibiotics against *S. aureus* strains as determined by broth microdilution assay following CLSI guidelines [25].

Antibiotic	MIC (µg/ml) against	MIC (µg/ml) against	MIC (µg/ml) against
	ATCC MSSA 29213	ATCC MRSA 33591	Clinical MRSA
Oxacillin	1 (S)	128 (R)	1024 (R)
Ciprofloxacin	0.5 (S)	0.5 (S)	32 (R)
Tetracycline	0.5 (S)	32 (R)	8 (R)
Gentamicin	0.5 (S)	0.5 (S)	128 (R)
Rifampicin	0.25 (S)	0.5 (R)	64 (R)

Note: (S) Strain susceptible to tested antibiotic.

(R) Strain resistant to tested antibiotic.

### Susceptibility Profile of Clinical MRSA to the Selected Antibiotics and to α-MSH by the Killing Assay

**Figure 1:**
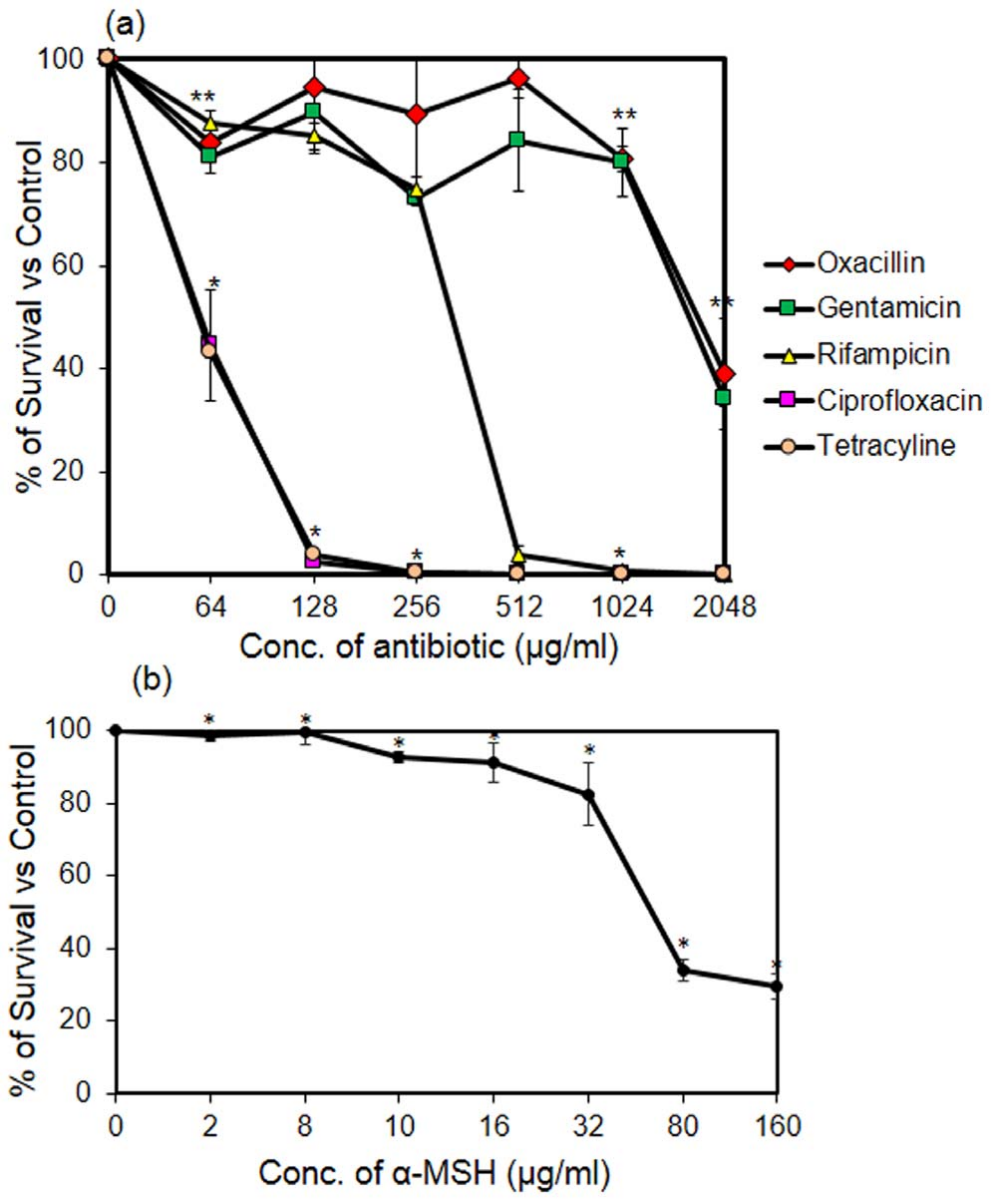
Determining bactericidal concentrations of each tested antibacterial agent. (a) Killing curves showing bactericidal activity of five different tested antibiotics against clinical MRSA isolate. Concentrations of each antibiotic were selected from 0 to 2048 µg/ml. Symbols; Oxacillin (red diamond), Gentamicin (green square), Rifampicin (yellow triangle), Ciprofloxacin (pink square) and Tetracycline (orange circle). (b) Bactericidal activity of α-MSH (2–160 µg/ml) against clinical MRSA isolate. The killing assay was done in triplicate and repeated on three different occasions. *p value ≤0.001, **p value ≤0.01, ***p value ≤0.05.

The killing activity of all selected antibiotics (at concentrations ranging from 64 to 2048 µg/ml) and α-MSH (2 to 160 µg/ml) was assessed against the clinical MRSA isolate. Taking into account the varying action time of the different chosen antibiotics, the antibiotic treatment was done for 2 h to allow sufficient time for both the slow-acting (i.e., OX) and fast-acting antibiotics (i.e., CF). Fig. 1a shows the percentage survival of bacteria treated with each selected antibiotic alone. Similarly, Fig. 1b shows the percentage survival of *S. aureus* cells treated with α-MSH alone for 2 h. The concentrations at which an antibiotic showed 90% and 50% bacterial killing were taken as the MBC_90_ and MBC_50_ value, respectively, of that antibiotic. These values were determined from the killing curves of the tested antibiotics (Fig. 1a), and are summarized in Table 2. It is apparent from Fig. 1a and Table 2 that the clinical MRSA strain was highly resistant to all the selected antibiotics. The MBC_90_ values of CF, TC and RF were 128 µg/ml, 128 µg/ml and 512 µg/ml, respectively (Table 2). However, in case of OX and GM, MBC_90_ could not be achieved even at 2048 µg/ml. Similarly, as shown in Fig. 1b, the strain was poorly susceptible to α-MSH as no killing was observed up to 8 µg/ml of α-MSH, and only 71.7±3.3% killing was obtained at 160 µg/ml of α-MSH (p<0.001 when multiple data-set of % survival were compared among different concentrations of α-MSH). It is pertinent to note here that in our previous reports we observed >95% killing by α-MSH against *S. aureus* ATCC 29213 even at a concentration of 2 µg/ml [22], [23].

**Table 2:** Minimum bactericidal concentration (MBC_50_ and MBC_90_) values of selected antimicrobial agents when used alone and in the presence of 8 µg/ml of α-MSH against clinical MRSA strain.

Antibiotic	MBC_50/_MBC_90_ (µg/ml) ofantibiotic alone	MBC_50/_MBC_90_ (µg/ml) of antibioticin the presence of α-MSH	Fold reduction in MBC values(MBC_50/_MBC_90_) of antibiotic whencombined with α-MSH
Oxacillin	2048/>2048	4/>32	512/NA*
Ciprofloxacin	64/128	2/16	32/8
Tetracycline	64/128	2/32	32/4
Gentamicin	2048/>2048	2/32	1024/>64
Rifampicin	>256/512	8/>32	>32/NA*

Note: Fold reduction = (MBC_50_ of antibiotic alone/MBC_50_ in presence of α-MSH).

NA*means MBC_90_ not achieved.

### Synergistic Interaction of α-MSH with Selected Conventional Antibiotics

**Figure 2:**
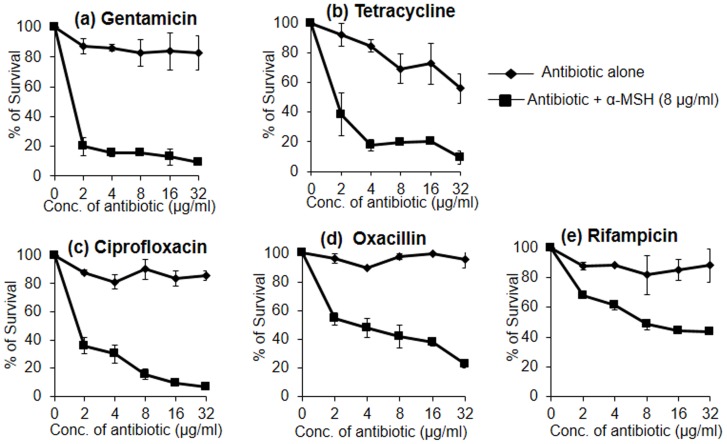
Killing curves of each of antibiotic alone and in presence of α-MSH against clinical MRSA isolate. (a) Gentamicin, (b) Tetracycline, (c) Ciprofloxacin, (d) Oxacillin, and (e) Rifampicin. Symbols; antibiotic alone (diamond) and antibiotic+α-MSH (8 µg/ml) (square). Experiments were repeated on three independent days. p value ≤0.05 (when multiple comparisons were done among % survival data-sets of different concentrations of same antibiotic with and without α-MSH).

The synergistic potential of α-MSH with the 5 different antibiotics was determined by the killing assay. The data with each antibiotic alone, and in the presence of 8 µg/ml of α-MSH is presented in Fig. 2. A remarkable increase in the staphylocidal activity of each antibiotic was observed when combined with α-MSH. Of the 5 antibiotics tested, GM (Fig. 2a), TC (Fig. 2b) and CF (Fig. 2c) demonstrated >90% killing in presence of α-MSH, whereas, the killing activity of the above antibiotics was <20% when they were administered alone in the same range of concentrations. For example, 2 to 32 µg/ml of GM alone showed only 13.7±4.9% to 16.8±11.5% bacterial killing; while at the same concentrations it showed 80.1±6.2% to 91.0±1.2% killing when used in combination with 8 µg/ml of α-MSH (Fig. 2a). Similarly, 2 to 32 µg/ml of OX alone showed 3.7±3.4% to 4.5±5.8% killing (Fig. 2d), whereas, in the presence of 8 µg/ml α-MSH, it killed 45.7±4.0% to 77.1±2.9% cells (Fig. 2d). The combination of RF and α-MSH, however, could only achieve 56±0.2% killing (Fig. 2e). The change in bactericidal activity of antibiotics in the presence of α-MSH was statistically significant (p<0.05 when compared among data-sets of different doses of each antibiotic alone and in the presence of α-MSH). The MBC_50_ and MBC_90_ values of all antibiotics were also determined in the presence of α-MSH from the above mentioned killing data (Fig. 2) and are summarized in Table 2. A synergistic relation was obtained when α-MSH was paired with GM, CF and TC since a ≥4-fold reduction in the MBC_90_ value was observed for all three antibiotics. For instance, the MBC_90_ value of GM alone was >2048 µg/ml, while in combination with α-MSH it was 32 µg/ml, thus achieving a 64-fold reduction (Table 2). Similarly, TC showed 32-fold and 4-fold reductions in MBC_50_ and MBC_90_ value, respectively, and CF showed 32-fold and 8-fold reductions in MBC_50_ and MBC_90_ value, respectively, in the presence of α-MSH. Although there was a huge reduction in MBC_50_ values of OX (512-fold) and RF (64-fold) in the presence of α-MSH, 90% killing could not be achieved even when they were combined with α-MSH.

### Impact of α-MSH on Bacterial DNA, RNA and Protein Synthesis

**Figure 3:**
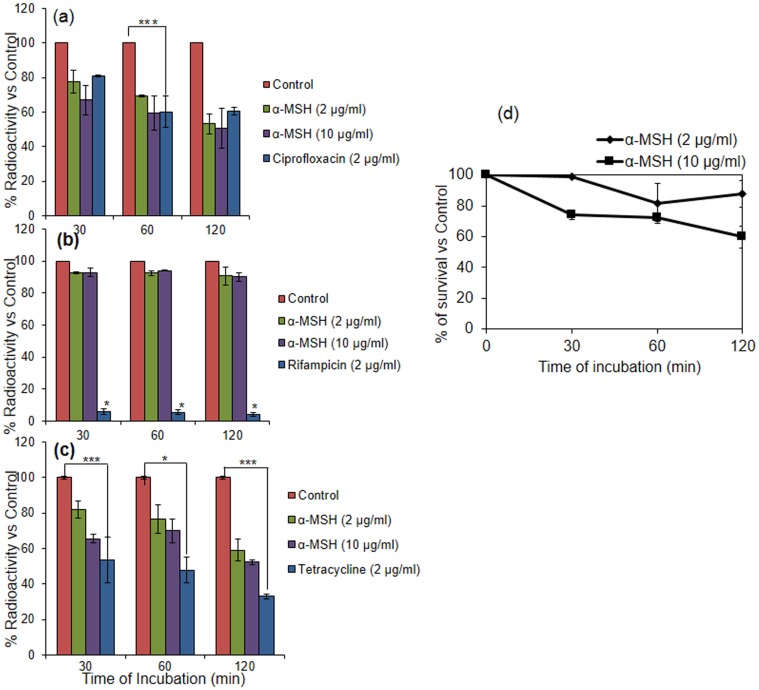
Impact of sub-lethal doses of α-MSH on DNA, RNA and protein synthesis of *S. aureus* ATCC 29213. (a) % of radioactivity of thymidine in untreated control (red), treated with 2 µg/ml α-MSH (green), 10 µg/ml α-MSH (purple) and 2 µg/ml of ciprofloxacin (blue); (b) % of radioactivity of uridine in control (red), treated with 2 µg/ml α-MSH (green), 10 µg/ml α-MSH (purple) and 2 µg/ml of rifampicin (blue); (c) % of radioactivity of leucine in control (red), treated with 2 µg/ml α-MSH (green), 10 µg/ml α-MSH (purple) and 2 µg/ml of tetracycline (blue); (d) killing kinetics of 2 µg/ml of α-MSH (diamond), and 10 µg/ml of α-MSH (square) against ∼10^8^ CFU/ml of *S. aureus* ATCC 29213. Experiments were done in duplicate and repeated on three independent days. *p value ≤0.001, **p value ≤0.01, ***p value ≤0.05.

To examine the effect of sub-lethal doses of α-MSH (2 and 10 µg/ml) on macromolecular synthesis of *S. aureus* ATCC 29213 cells, incorporation of radioactive precursor [methyl-^3^H] thymidine, [5-^3^H] uridine and [4,5-^3^H] leucine into DNA, RNA and protein, respectively, was observed over a period of 2 h and percent radioactivity of these precursors is presented in Fig. 3a–c. Additionally, ciprofloxacin, rifampicin and tetracycline treated samples (2 µg/ml each) were included as positive controls of DNA, RNA and protein synthesis inhibition, respectively [44]. As can be seen from Fig. 3a and Fig. 3c, a reduction in the incorporation of [methyl-^3^H] thymidine and [4,5-^3^H] leucine was observed in the α-MSH treated samples. For example, [methyl-^3^H] thymidine radioactivity showed a reduction from 100% (untreated control) to 77.6±6.8% and 67.0±8.5% when treated with 2 and 10 µg/ml of α-MSH, respectively, for 30 min. It further reduced to 53.2±5.9% and 50.8±11.6% after 2 h of treatment (Fig. 3a). Likewise, a 18±4.5% and 34.5±2.1% decrease in the incorporation of [4,5-^3^H] leucine was observed in samples treated with 2 and 10 µg/ml of α-MSH, respectively, for 30 min (Fig. 3c), and was further decreased by 40.9±6.3% and 47±1.4%, after 2 h treatment with α-MSH (Fig. 3c). The radioactive labeling assay indicated that there was inhibition of both DNA and protein synthesis in the bacterial cells on exposure to sub-lethal concentrations of α-MSH. However, less than a 10% decrease in the incorporation of [5-^3^H] uridine was observed in the α-MSH treated samples compared to the untreated controls, suggesting that α-MSH had only a marginal effect on RNA synthesis (<10%) (Fig. 3b).

To confirm that the chosen doses of α-MSH were not bactericidal, killing kinetics using the 2 and 10 µg/ml of α-MSH against ∼10^8^ CFU/ml of *S. aureus* ATCC 29213 were performed simultaneously, and the killing results are shown in Fig. 3d. The data showed that only ∼12% and 40±6.9% killing was observed in cells treated with 2 µg/ml and 10 µg/ml of α-MSH, respectively, over a period of 2 h.

### Effect of α-MSH Toxicity on Mouse Red Blood Cells (RBCs) and Fibroblast Cell Lines

**Figure 4:**
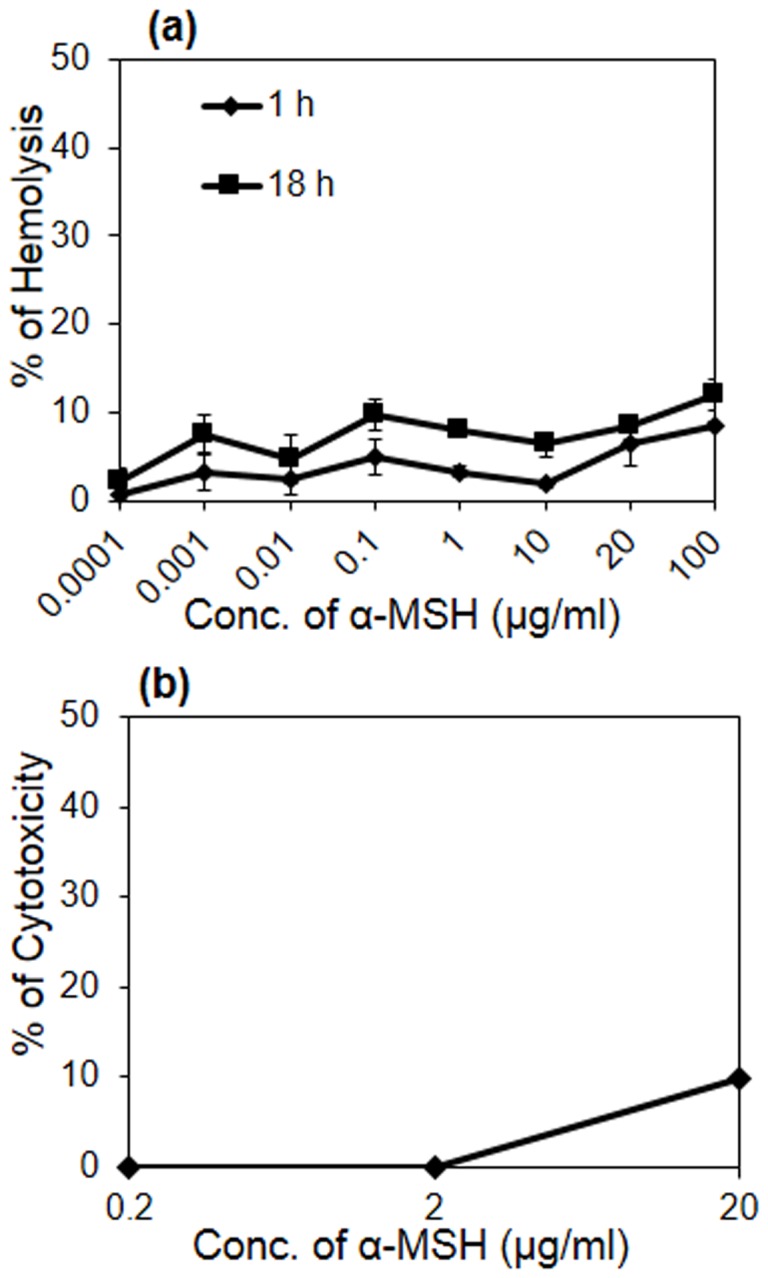
Cellular toxicity due to α-MSH. (a) Hemolytic effect of α-MSH (100 pg/ml to 100 µg/ml) on mice RBCs after 1 h (diamond) and 18 h (square) of incubation, (b) cytotoxic effect of α-MSH (0.2 µg/ml to 20 µg/ml) on the mouse fibroblast cell lines. Each assay was done in triplicate on two different days.

The hemolysis of mouse RBCs exposed to a range of α-MSH concentrations (100 pg/ml to 100 µg/ml) was examined (Fig. 4a). As can be seen from this figure, less than 10% hemolysis was observed after 1 h treatment with α-MSH even at a concentration of 100 µg/ml, and only ∼3% further increase in hemolysis was observed when exposure time was increased to 18 h. Fibroblast cell cytotoxicity due to α-MSH exposure was measured using MTT assay (Fig. 4b). The survival of cells was 100% upon exposure to 2 µg/ml of α-MSH, and a mere 9.8% loss in cell viability occurred on exposure to 20 µg/ml of α-MSH. Taken together, it is evident that α-MSH has negligible hemolytic and cytotoxic effects at concentrations well above the dose required for its antibacterial effect.

## Discussion

We face a grave health risk due to the failure of existing antibiotics in treating multidrug resistant strains of *S. aureus* both in the hospital and community settings [45], [46], [47]. Enormous efforts have been made worldwide to develop novel sustainable antibacterial agents. Although HDPs have the potential to be developed as a new class of therapeutics [48], their toxicity at the required doses is a major drawback. To address this problem, among the different strategies, the combination of HDPs with conventional antibiotics is receiving wide attention [49], [50].

In our previously published studies, we demonstrated the strong antibacterial activity of a neuropeptide, α-MSH, against various strains of *S. aureus*, including the MRSA strains [21], [22]. We further proved that α-MSH caused membrane damage leading to leakage of cellular content and depolarization, and eventual cell-killing. In the present study, we explored the *in vitro* synergistic potential of α-MSH with GM, CF, TC, OX, and RF against a clinical isolate of *S. aureus* identified as MRSA (ST239-SCC*mec* III). The ST239 has been identified as a major cause of MRSA infections in Asian healthcare settings, including India [24], [42], [51]. The antibiotics were chosen because they belonged to different classes of antimicrobial agents and their modes of action are quite different from one another. For example, GM and TC primarily act on protein synthesis whereas CF targets DNA replication [44], [52]. RF inhibits DNA-dependent RNA polymerase activity in bacterial cells [53] and OX belongs to the β-lactam family, interferes with bacterial cell wall synthesis by attachment with penicillin binding protein [44].

Our present study strongly suggests that α-MSH acts synergistically with GM, CF and TC. For example, 2 h incubation with 32 µg/ml of GM, CF and TC showed 17%, 14% and 45% killing of *S. aureus* cells, respectively. When used in combination with 8 µg/ml α-MSH, killing activity increased to 91%, 93% and 90%, respectively (Fig. 2a, b, & c). The fold reduction in MBC_90_ values of GM, CF and TC was >64, 8, and 4, respectively, when used in combination with α-MSH. This suggests that 90% killing was achieved with a much lower dose of these antibiotics when used in combination with α-MSH. Synergy was robust with the combination of GM and α-MSH with a >64-fold reduction in MBC_90_ (Table 2). GM is effective against staphylococcal infection but exhibits dose-limiting toxicities [54]. Since the addition of α-MSH with GM lowered the required dose of the antibiotic, it could be an alternative and effective treatment for staphylococcal infections. The addition of α-MSH to OX substantially increased the bacterial killing (Fig. 2d). However, >90% bactericidal activity could not be achieved using this combination. This may be attributed to the fact that the strain used was highly resistant to OX (MIC = 1024 µg/ml) and the maximum dose of OX tested in the study was 32 µg/ml. Synergy may be observed upon using a higher dose of OX and increasing the incubation time. The combination of α-MSH and RF was only additive (Fig. 2e) as only ∼45% increase in killing of RF was obtained when combined with α-MSH.

We next sought to further delineate the mechanism of action of α-MSH on *S. aureus* cells. We have previously shown that membrane permeabilization was a major mechanism of staphylocidal action of α-MSH. However, other targets could not be ruled out [22]. This was because a time lag was observed between bacterial cell death (occurring within 15 minutes of peptide exposure) and substantial membrane damage (occurring between 30–120 min after α-MSH exposure) [23]. In an attempt to understand whether membrane damage due to α-MSH exposure was the lone cause of cell death, or whether like other HDPs (LL−37 and human α-defensin) [55], α-MSH also caused pleiotropic intracellular effects, we evaluated the impact of α-MSH on DNA, RNA and protein synthesis. Radioactive whole-cell labeling assays showed 53% and 47% reduction in the incorporation of thymidine and leucine into DNA and protein, respectively, in the presence of sub-inhibitory doses of α-MSH. In contrast, only a marginal (<10%) reduction in the incorporation of uridine into RNA (Fig. 3b.) was observed in α-MSH treated cells. Taken together, these observations indicate that besides membrane damaging properties; α-MSH possesses the capability of hampering DNA replication and protein synthesis of *S. aureus* ATCC 29213, directly or indirectly, with little effect on RNA synthesis.

These observations have important implications in understanding the synergy observed between the antibiotics and the peptide. It has been reported that the membrane permeabilizing activity of HDPs can increase the uptake of antibiotics in the resistant *S. aureus* strains, thereby decreasing the effective antibiotic dose [14], [54]–[57]. As observed by others [56], [57], the ability of α-MSH to increase membrane permeability may have facilitated the entry of all the antibiotics studied here, thus increasing their efficacy in bacterial killing. The more pronounced synergistic activity in the case of CF, GM, and TC may be due to a mechanistic analogy between these antibiotics and α-MSH. For instance, CF targets DNA replication and GM and TC target protein synthesis to cause their antibacterial action. This study showed the diminishing effect of α-MSH on DNA and protein synthesis. The common killing mechanism (either inhibition of protein synthesis or inhibition of DNA synthesis) along with other known (like membrane damaging ability of the peptide) or unknown mechanisms make the combination of GM or TC or CF with α-MSH synergistic.

The combination of OX with α-MSH was also very promising. OX primarily acts on the staphylococcal cell wall and α-MSH causes rapid changes in cell membrane permeability. Despite their different targets, each agent may complement the effect of the other, leading to substantial increase in bacterial cell death. On the contrary, a moderate increase in the effect of RF and α-MSH combination may be due to the absence of a common mechanism of bactericidal action. This pair appeared to act additively rather than synergistically. As already pointed out, the membrane permeabilizing property of α-MSH probably helps RF in entering the cells and hence an increase in antibacterial activity of RF was obtained in presence of α-MSH.

The lack of any appreciable mammalian cell cytotoxiciy and hemolytic activity (Fig. 4) as a result of α-MSH exposure is important since it further enhances its possible medical applications. Our previous studies had already suggested that α-MSH was a prospective candidate to be developed as an anti-staphylococcal agent. The findings of the present study, using a highly resistant staphylococcal strain, have further broadened the therapeutic potential of α-MSH.

We would also like to add a word of caution here. Mechanisms of resistance in *S. aureus* are multifactorial and vary significantly from strain to strain. Emergence of varied clonal complexes among MRSA strains indicates its extraordinary ability to adapt and develop resistance [45], [47]. Therefore, more clinical strains from different genetic backgrounds need to be studied before α-MSH may be used against all strains of *S. aureus*. Detailed *in vivo* studies will also be required before this combination therapy moves from the bench to the bedside. Nevertheless, these results raise interesting possibilities for future studies.

In summary, we have shown for the first time that α-MSH acts synergistically and additively with various classes of conventional antibiotics by drastically reducing the required dose of the antibiotics as well as the peptide itself. In the long run, combination therapy involving antibiotics and antimicrobial peptides may perhaps be a viable strategy to improve the efficacy of treatment in a cost-effective manner.

## References

[1] Tiwari HK, Sapkota D, Sen MR (2008) High prevalence of multidrug-resistant MRSA in a tertiary care hospital of northern India. Infect Drug Resist 1: 57–61. 21694881 10.2147/idr.s4105 PMC3108723

[2] Otter JA, French GL (2008) The emergence of community-associated methicillin-resistant *Staphylococcus aureus* at a London teaching hospital, 2000–2006. Clin Microbiol Infect 14: 670–676. 18558939 10.1111/j.1469-0691.2008.02017.x

[3] Klevens RM, Morrison MA, Nadle J, Petit S, Gershman K, et al (2007) Invasive methicillin-resistant *Staphylococcus aureus* infections in the United States. JAMA 298: 1763–1771. 17940231 10.1001/jama.298.15.1763

[4] Fry DE, Barie PS (2011) The changing face of *Staphylococcus aureus*: a continuing surgical challenge. Surg Infect 12: 191–203. 10.1089/sur.2011.068 21812657

[5] Wong A, Reddy SP, Smyth DS, Aguero-Rosenfeld ME, Sakoulas G, et al (2010) Polyphyletic emergence of linezolid-resistant staphylococci in the United States. Antimicrob Agents Chemother 54: 742–748. 19933808 10.1128/AAC.00621-09 PMC2812165

[6] Yang SJ, Xiong YQ, Dunman PM, Schrenzel J, François P, et al (2009) Regulation of mprF in daptomycin-nonsusceptible *Staphylococcus aureus* strains. Antimicrob Agents Chemother 53: 2636–2637. 19289517 10.1128/AAC.01415-08 PMC2687189

[7] Jawetz E (1968) The use of combinations of antimicrobial drugs. Annu Rev Pharmacol 8: 151–170. 4875390 10.1146/annurev.pa.08.040168.001055

[8] Krogstad DJ, Moellering RC, Jr. (1980) Combinations of antibiotics; mechanisms of interaction against bacteria. In: V Lorian editor. Antibiotics in Laboratory Medicine. Baltimore. The Williams & Wilkins Co. 300–309.

[9] Moreillon P, Que YA (2004) Infective endocarditis. Lancet 363: 139–149. 14726169 10.1016/S0140-6736(03)15266-X

[10] Entenza JM, Veloso TR, Vouillamoz J, Giddey M, Majcherczyk P, et al (2011) *In vivo* synergism of ceftobiprole and vancomycin against experimental endocarditis due to vancomycin-intermediate *Staphylococcus aureus* . Antimicrob Agents Chemother 55: 3977–3984. 21730114 10.1128/AAC.00402-11 PMC3165304

[11] Credito K, Lin G, Appelbaum PC (2007) Activity of daptomycin alone and in combination with rifampin and gentamicin against *Staphylococcus aureus* assessed by time-kill methodology. Antimicrob Agents Chemother 51: 1504–1507. 17220402 10.1128/AAC.01455-06 PMC1855505

[12] Zasloff M (2002) Antimicrobial peptides of multicellular organisms. Nature 415: 389–395. 11807545 10.1038/415389a

[13] Catania A, Colombo G, Rossi C, Carlin A, Sordi A, et al (2006) Antimicrobial properties of α-MSH and related synthetic melanocortins. Scientific World Journal 6: 1241–1246. 17028769 10.1100/tsw.2006.227 PMC5917254

[14] Hancock REW, Patrzykat A (2002) Clinical development of cationic antimicrobial peptides: from natural to novel antibiotics. Curr Drug Targets Infect Disord 2: 79–83. 12462155 10.2174/1568005024605855

[15] Yount NY, Yeaman MR (2012) Emerging themes and therapeutic prospects for anti-infective peptides. Annu Rev Pharmacol Toxicol 52: 337–360. 22235859 10.1146/annurev-pharmtox-010611-134535

[16] Chen X, Niyonsaba F, Ushio H, Okuda D, Nagaoka I, et al (2005) Synergistic effect of antibacterial agents human β-defensins, cathelicidin LL−37 and lysozyme against *Staphylococcus aureus* and *Escherichia coli* . J Dermatol Sci 40: 123–132. 15963694 10.1016/j.jdermsci.2005.03.014

[17] van der Linden DS, Short D, Dittmann A, Yu PL (2009) Synergistic effects of ovine-derived cathelicidins and other antimicrobials against *Escherichia coli* O157: H7 and *Staphylococcus aureus* 1056 MRSA. Biotechnol Lett 31: 1265–1267. 19396584 10.1007/s10529-009-0010-9

[18] Park Y, Park SN, Park SC, Shin SO, Kim JY, et al (2006) Synergism of leu-Lys antimicrobial peptides and chloramphenicol against bacterial cells. Biochim Biophys Acta 1764: 24–32. 16344012 10.1016/j.bbapap.2005.10.019

[19] Pankey GA, Ashcraft DS (2011) Detection of synergy using the combination of polymyxin B with either meropenem or rifampin against carbapenemase-producing *Klebsiella pneumonia* . Diagn Microbiol Infect Dis 70: 561–564. 21767715 10.1016/j.diagmicrobio.2011.05.003

[20] Rishi P, Preet S, Bharrhan S, Verma I (2011) *In vitro* and *in vivo* synergistic effects of cryptidin 2 and ampicilin against *Salmonella* . Antimicrob Agents Chemother 55: 4176–4182. 21690282 10.1128/AAC.00273-11 PMC3165299

[21] Shireen T, Singh M, Dhawan B, Mukhopadhyay K (2012) Characterization of cell membrane parameters of clinical isolates of *Staphylococcus aureus* with varied susceptibility to Alpha-melanocyte stimulating hormone. Peptides 37: 334–339. 22835936 10.1016/j.peptides.2012.05.025

[22] Madhuri, Shireen T, Venugopal SK, Ghosh D, Gadepalli R, et al (2009) *In vitro* antimicrobial activity of alpha-melanocyte stimulating hormone against major human pathogen *Staphylococcus aureus* . Peptides 30: 1627–35. 19560499 10.1016/j.peptides.2009.06.020

[23] Singh M, Mukhopadhyay K (2011) C-terminal amino acids of alpha-melanocyte stimulating hormone are requisite for its antibacterial activity against *Staphylococcus aureus.* . Antimicrob Agents Chemother 55: 1920–1929. 21282427 10.1128/AAC.00957-10 PMC3088230

[24] Gadepalli R, Dhawan B, Kapil A, Sreenivas V, Jais M, et al (2009) Clinical and molecular characteristics of nosocomial methicillin-resistant *Staphylococcus aureus* and soft tissue skin isolates from three Indian hospitals. J Hosp Infect 73: 253–263. 19782432 10.1016/j.jhin.2009.07.021

[25] Clinical and Laboratory Standards Institute (2009) Performance standards for antimicrobial susceptibility testing; Nineteenth informational supplement. CLSI Document M100-S19. CLSI, Wayne, PA.

[26] Oliveira DC, de Lencastre H (2002) Multiplex PCR strategy for rapid identification of structural types and variants of the *mec* element in methicillin-resistant *Staphylococcus aureus* . Antimicrob Agents Chemother 46: 2155–2161. 12069968 10.1128/AAC.46.7.2155-2161.2002 PMC127318

[27] Enright MC, Day NP, Davies CE, Peacock SJ, Spratt BG (2000) Multilocus sequence typing for characterization of methicillin-resistant and methicillin-susceptible clones of *Staphylococcus aureus* . J Clin Microbiol 38: 1008–1015. 10698988 10.1128/jcm.38.3.1008-1015.2000 PMC86325

[28] Traczewski MM, Goldmann DA, Murphy P (1983) *In vitro* activity of rifampin in combination with oxacillin against *Staphylococcus aureus* . Antimicrob Agents Chemother 23: 571–576. 6859835 10.1128/aac.23.4.571 PMC184703

[29] Schafer JA, Hovde LB, Rotschafer JC (2006) Consistent rates of kill of *Staphylococcus aureus* by gentamicin over a 6-fold clinical concentration range in an *in vitro* pharmacodynamic model (IVPDM). J Antimicrob Chemother 58: 108–111. 16735429 10.1093/jac/dkl216

[30] Sanfilippo CM, Hesje CK, Haas W, Morris TW (2011) Topoisomerase mutations that are associated with high-level resistance to earlier fluoroquinolones in *Staphylococcus aureus* have less effect on the antibacterial activity of besifloxacin. Chemotherapy 57: 363–371. 21996946 10.1159/000330858

[31] Kronvall G, Karlsson I, Walder M, Sorberg M, Nilsson LE (2006) Epidemiological MIC cut-off values for tigecycline calculated from Etest MIC values using normalized resistance interpretation. J Antimicrob Chemother 57: 498–505. 16410264 10.1093/jac/dki489

[32] Turner J, Cho Y, Dinh NY, Waring AJ, Lehrer RI (1998) Activities of LL−37, a cathelin-associated antimicrobial peptide of human neutrophils. Antimicrob Agents Chemother 42: 2206–2214. 9736536 10.1128/aac.42.9.2206 PMC105778

[33] Javadpour MM, Juban MM, Lo WC, Bishop SM, Alberty JB, et al (1996) De novo antimicrobial peptides with low mammalian cell toxicity. J Med Chem 39: 3107–3113. 8759631 10.1021/jm9509410

[34] Pogwizd SM, Lerner SA (1976) *In vitro* activity of gentamicin, amikacin, and netilmicin alone and in combination with carbenicillin against *Serratia marcescens.* . Antimicrob Agents Chemother 10: 878–884. 795373 10.1128/aac.10.6.878 PMC429858

[35] Cohen MA, Huband MD, Yoder SL, Gage JW, Roland GE (1998) Bacterial eradication by clinafloxacin, CI−990, and ciprofloxacin employing MBC test, *in vitro* time–kill and *in vivo* time–kill studies. J Antimicrob Chemother 41: 605–614. 9687098 10.1093/jac/41.6.605

[36] Minuth JN, Holmes TM, Musher DM (1974) Activity of tetracycline, doxycycline, and minocycline against methicillin -susceptible and -resistant staphylococci. Antimicrob Agents Chemother 6: 411–414. 4157335 10.1128/aac.6.4.411 PMC444661

[37] Tuazon CU, Lin MYC, Sheagren JN (1978) *In vitro* activity of rifampin alone and in combination with nafcillin and vancomycin against pathogenic strains of *Staphylococcus aureus.* . Antimicrob Agents Chemother 13: 759–761. 666300 10.1128/aac.13.5.759 PMC352328

[38] Yenugu S, Hamil KG, Radhakrishnan Y, French FS, Hall SH (2004) The androgen-regulated epididymal sperm-binding protein, human β-Defensin 118 (DEFB118) (Formerly ESC42), is an antimicrobial β-defensin. Endocrinology 145: 3165–3173. 15033915 10.1210/en.2003-1698

[39] Menzel TM, Tischer M, François P, Nickel J, Schrenzel J, et al (2011) Mode-of-action studies of the novel bisquaternary bisnaphthalimide MT02 against *Staphylococcus aureus.* . Antimicrob Agents Chemother 55: 311–320. 20937782 10.1128/AAC.00586-10 PMC3019648

[40] Chou HT, Wen HW, Kuo TY, Lin CC, Chen WJ (2010) Interaction of cationic antimicrobial peptides with phospholipid vesicles and their antibacterial activity. Peptides 31: 1811–1820. 20600422 10.1016/j.peptides.2010.06.021

[41] Khanavi M, Gheidarloo R, Sadati N, Ardekani MR, Nabavi SM, et al (2010) Cytotoxicity of fucosterol containing fraction of marine algae against breast and colon carcinoma cell line. Biol Res 43: 31–37. 22438665 10.4103/0973-1296.93327 PMC3307205

[42] Arakere G, Nadig S, Ito T, Ma XX, Hiramatsu K (2009) A novel type-III staphylococcal cassette chromosome *mec* (SCC*mec*) variant among Indian isolates of methicillin-resistant *Staphylococcus aureus* . FEMS Microbiol Lett 292: 141–148. 19187210 10.1111/j.1574-6968.2008.01482.x

[43] Turnidge J, Paterson DL (2007) Setting and revising antibacterial susceptibility breakpoints. Clin Microbiol Rev 20: 391–408. 17630331 10.1128/CMR.00047-06 PMC1932754

[44] Tenover FC (2006) Mechanisms of antimicrobial resistance in bacteria. The Am J Med 119: S3–10. 10.1016/j.amjmed.2006.03.011 16735149

[45] Wu K, Zhang K, McClure J, Zhang J, Schrenzel J, et al (2013) A correlative analysis of epidemiologic and molecular characteristics of methicillin-resistant *Staphylococcus aureus* clones from diverse geographic locations with virulence measured by a *Caenorhabditis elegans* host model. Eur J Clin Microbiol Infect Dis 32: 33–42. 22898726 10.1007/s10096-012-1711-x PMC3545200

[46] Boucher HW, Corey GR (2008) Epidemiology of methicillin-resistant *Staphylococcus aureus.* . Clin Infect Dis 46: S344–S349. 18462089 10.1086/533590

[47] Chambers HF, DeLeo FR (2009) Waves of resistance: *Staphylococcus aureus* in the antibiotic era. Nat Rev Microbiol 7: 629–641. 19680247 10.1038/nrmicro2200 PMC2871281

[48] Rice LB (2008) Federal funding for the study of antimicrobial resistance in nosocomial pathogens: no ESKAPE. J Infect Dis 197: 1079–1081. 18419525 10.1086/533452

[49] Leszczyńska K, Namiot A, Janmey PA, Bucki R (2010) Modulation of exogenous antibiotic activity by host cathelicidin LL−37. Acta Pathologica, Microbiologica et Immunologica Scandinavica 118: 830–836. 10.1111/j.1600-0463.2010.02667.x PMC3386844 20955455

[50] Sakoulas G, Bayer AS, Pogliano J, Tsuji BT, Yang SJ (2012) Ampicillin enhances daptomycin- and cationic host defense peptide-mediated killing of ampicillin- and vancomycin-resistant *Enterococcus faecium.* . Antimicrob Agents Chemother 56: 838–844. 22123698 10.1128/AAC.05551-11 PMC3264218

[51] Smyth DS, McDougal LK, Gran FW, Manoharan A, Enright MC, et al (2009) Population structure of a hybrid clonal group of methicillin-resistant *Staphylococcus aureus* ST239-MRSA-III. PLoS One 5: e8582. 10.1371/journal.pone.0008582 PMC2797301 20062529

[52] Drlica K, Zhao X (1997) DNA gyrase, topoisomerase IV, and the 4-quinolones. Microbiol Mol Bio Rev 61: 377–392. 9293187 10.1128/mmbr.61.3.377-392.1997 PMC232616

[53] Campbell EA, Korzheva N, Mustaev A, Murakami K, Nair S, et al (2001) Structural mechanism for rifampicin inhibition of bacterial RNA polymerase. Cell 104: 901–912. 11290327 10.1016/s0092-8674(01)00286-0

[54] Lopez-Novoa JM, Quiros Y, Vicente L, Morales AI, Lopez-Hernandez F (2011) New insights into the mechanism of aminoglycoside nephrotoxicity: an integrative point of view. Kidney Int 79: 33–45. 20861826 10.1038/ki.2010.337

[55] Xiong YQ, Yeaman MR, Bayer AS (1999) *In vitro* antibacterial activities of platelet microbicidal protein and neutrophil defensin against *Staphylococcus aureus* are influenced by antibiotics differing in mechanism of action. Antimicrob Agents Chemother 43: 1111–1117. 10223922 10.1128/aac.43.5.1111 PMC89119

[56] Yenugu S, Narmadha G (2010) The human male reproductive tract antimicrobial peptides of HE2 family exhibit potent synergy with standard antibiotics. J Pept Sci 16: 337–341. 20552564 10.1002/psc.1246

[57] Nadrah K, Strle F (2011) Antibiotic combinations with daptomycin for the treatment of *Staphylococcus aureus* infections. Chemother Res Pract 2011: 1–10. 10.1155/2011/619321 PMC3265245 22312555

